# Global 0.05° Grid-Based Dataset of Keyhole Imagery with Spatio-Temporal Indicators (1960–1984)

**DOI:** 10.1038/s41597-026-06866-4

**Published:** 2026-02-17

**Authors:** Tao Wang, Xinle Zhang, Mulin Shan, Mingyuan Deng, Jiaheng Wang, Huanjun Liu, Hao Li, Jinyu Sun

**Affiliations:** 1 School of Geography and Environment, Liaocheng University, Liaocheng, 252059 China; 2 College of Information Technology, Jilin Agricultural University, Changchun, 130118 China; 3 State Key Laboratory of Black Soils Conservation and Utilization, Northeast Institute of Geography and Agroecology, Chinese Academy of Sciences, Changchun, 130102 China

**Keywords:** Environmental sciences, Ecology

## Abstract

The American satellite reconnaissance program (Keyhole imagery) is serving as a significant data source for geoscience research because of its high-resolution and early temporal coverage, while lack of spatial and temporal description of its uneven distribution could hinder researchers from selecting/accessing appropriate the Keyhole images. Here we introduce a global grid–based dataset that organizes declassified U.S. Keyhole imagery (1960–1984) for direct reuse, built on a global equal-area sinusoidal grid. This dataset standardizes scene metadata and provides indicators designed to inform study design and data integration: coverage count (how often a place was imaged), unique acquisition dates (temporal sampling richness), first/last observation year (temporal bounds), observation span (duration), peak observation year and a three-year window (temporal concentration), resolution class (C1–C3), temporal-coverage class across five five-year intervals, and resolution-coverage class (A–G) for multi-scale availability. This dataset enables users to quickly locate usable scenes, assess temporal suitability, combine historical images with modern satellites, and determine which non-free images to purchase if free images were unsuitable for their research.

## Background & Summary

Beginning in the early 1960s, the United States operated a series of reconnaissance satellite missions under the codename *Keyhole*^1^. These missions, designed primarily for strategic reconnaissance and intelligence purposes during the Cold War, collected imagery at resolutions ranging from ten meters to sub-meter levels—representing the most advanced optical surveillance technology of that time^2,3^. The early generations relied on film-return systems, which required physical capsule recovery and thus limited both mission duration and temporal continuity^4^. As a result, while the archive constitutes a remarkable technological achievement, its coverage was inherently shaped by technical design constraints and military priorities. Following phased declassification in 1995, 2002, and 2012, different portions of the Keyhole archive became publicly available. Parts of Keyhole archive are free download, and others require purchase for 30$ per image. Once purchased by any user, an image becomes publicly freely accessible to the public, so the number of free-download images is increasing over time. Given that the earliest civil satellite programs as Landsat MSS only began in 1972 with coarse spatial resolutions of 30–60 m^5,6^, the Keyhole archive remains the sole global dataset providing high-resolution imagery of the Earth’s surface from the 1960s to the 1980s.

Because of their high spatial resolution—ranging from sub-meter to decameter levels—and their unique temporal coverage spanning the 1960s to the 1980s, Keyhole images have become indispensable for historical Earth observation. Combined with modern remote sensing images, Keyhole images can effectively reveal deforestation^7,8^, erosion process^9^, agricultural expansion, and urban growth^10^ that shaped landscapes. Archaeology is one of the most prominent domains of application since Keyhole imagery has been employed to map ancient irrigation systems^11^, fortifications^12^, and settlement patterns^13^ across the Middle East and North Africa. KH-9 stereo pairs have been processed into historical digital elevation models (DEMs), enabling estimates of ice volume loss and glacier retreat in the Himalayas^14,15^, and more than one third of surging glaciers were identified after incorporating KH-9 DEMs than using only COP30 DEMs^16^. Similarly, researchers have used CORONA data to evaluate river migration^17^, coastal change^18^, and delta evolution^19^, documenting the geomorphic processes that affect water resources and hazard exposure^20^. Recent advances show that standardized preprocessing and classification workflows can render Keyhole imagery a reliable and reproducible resource for long-term land use mapping, achieving high accuracies when combined with ancillary terrain information^21^. These diverse applications have transitioned declassified Keyhole imagery from a military reconnaissance dataset to a versatile scientific resource, bridging the observational gap in Earth system research during the mid-20th century and providing a unique temporal reference for understanding environmental and cultural change.

Despite its significance, the spatial and temporal distribution of Keyhole imagery is highly uneven. Unlike modern Earth observation satellites such as Landsat ETM or SPOT that operate in repetitive orbital cycles with balanced ground coverage^22^, Keyhole film-return missions had short on-orbit lifetimes and irregular spatial footprints, resulting in highly uneven coverage. Imagery acquisition was heavily concentrated over geopolitical hotspots and areas of strategic interest—such as Soviet industrial complexes, Chinese nuclear facilities, and military installations across Europe and the Middle East^23^. Besides, the free-download images only represent a subset of the full declassified Keyhole archive, so its spatial-temporal distribution differs from that of the full dataset^24^. Researchers estimated the number of additional non-free images required to achieve consistent triple coverage for meter-level imagery across China and calculated associated costs^25^, which would require 100–150 thousand US dollars with 30$ per image. These analyses illustrate how assessing heterogeneity is not only critical for identifying feasible regions for land-use change detection, but also provides practical guidance for planning supplementary data acquisition and budgeting.

Although regional studies revealed the spatial heterogeneity of Keyhole imagery, comprehensive global assessments remain lacking. The quantified revisit intervals and coverage patterns of the PlanetScope constellation^26^ proved that analyzing the global distribution of remote sensing imagery could facilitate both the clarification of dataset characteristics and the expansion of its applications. In particular, systematic evaluation of satellite imagery’ spatial coverage, temporal depth, and resolution composition is necessary to understand where and how these data can be most effectively used^27^. Such evaluation not only supports multi-temporal land-surface change detection but also informs the cost-effectiveness of acquiring commercial Keyhole imagery in underrepresented regions.

In this work we developed a global dataset (KRIST, Keyhole Reconnaissance Imagery Spatio-Temporal coverage^28^, 10.6084/m9.figshare.30017944) that characterizes the spatio-temporal coverage of Keyhole imagery from 1960–1984 including free and full dataset. Built on a regular 0.05° global grid of more than 25 million points, the dataset integrates multiple resolution levels of Keyhole imagery, ranging from tens-meter to meter scale. Their spatial and temporal coverage through standardized indicators, including acquisition frequency, number of unique observation dates, observation time span, and peak observation years were characterized. To account for temporal heterogeneity, the archive was divided into consecutive five-year intervals from 1960 to 1984. Their combinations were summarized into broader coverage classes, enabling the identification of regions with concentrated, evenly distributed, or fragmented temporal observations. Provided in an open and interoperable GeoPackage, netCDF and HDF5 format, this dataset delivers the first globally consistent framework for evaluating Keyhole imagery coverage across space, time, and resolution. KRIST is intended to lower barriers to data access, offer a basis for multi-temporal Earth system studies, and provide a reference for identifying regions where supplementary data may be most valuable.

## Data Acquisition and Organization

### Data sources

KRIST dataset presented in this study is derived from the declassified archive of U.S. reconnaissance satellite missions, collectively known under the codename *Keyhole*, operated between the early 1960s and mid-1980s. These missions, including the Corona, Argon, Lanyard, and Hexagon series, represent the earliest generation of high-resolution satellite imagery. Depending on mission type and orbital configuration, the spatial resolution of the images ranged from sub-meter to more than ten meters. For instance early Corona missions delivered imagery at 2–7 m resolution, and later Hexagon satellites achieved sub-meter resolution^29^.

### Metadata processing

Both of free and full Keyhole imagery footprints including attributes were retrieved from the United States Geological Survey (USGS) EarthExplorer platform (https://earthexplorer.usgs.gov/). Particularly the free dataset was downloaded using the *“free download”* filter. There were 62,715 and 1,429,665 scenes in free and full datasets globally between 1960 and 1984. The imagery is distributed across three datasets released in different stages of declassification. The first dataset (Declass 1, D1) includes early reconnaissance missions such as KH-1, KH-2, KH-3, KH-4, KH-4A, KH-4B, KH-5, and the KH-6, operated between 1960 and 1972 (10.5066/F78P5XZM). The second dataset (Declass 2, D2) contains the KH-7 system with resolutions of approximately 2–4 feet, together with the low-resolution KH-9 system (KH-9L, 20–30 feet), acquired between 1971 and 1984 (10.5066/F74X5684). The third dataset (Declass 3, D3) consists of the high-resolution KH-9 series (KH-9H, 2–4 feet), collected between 1971 and 1984 (10.5066/F7WD3Z10). The duplicate KH-9L images (20~30 feet) in D3 which are already included in D2 are deleted (294 images in free dataset and 943 images in full dataset).

Because the attribute tables of D1, D2 and D3 datasets are heterogeneous, a harmonization process was implemented to standardize the metadata and enable cross-dataset comparison. USGS-provided records contain diverse fields such as mission codes, qualitative resolution descriptors, tile identifiers, and acquisition details, depending on the collection. To unify these records, a minimal but consistent schema was defined, retaining the following attributes: *Entity ID, Acquisition date*, *Mission*, *Satellite*, *Resolution Class (res_clas)*, *Polygon ID*, and *Metadata URL*. *Acquisition dates* were normalized to the ISO 8601 standard, and *Entity ID* provides a unique reference to each scene. The *Mission* field identifies the operational number within each satellite series (e.g., KH-7 includes more than twenty operations such as 4001, 4002). The *Satellite* field specifies the broader reconnaissance system (e.g., KH-7, KH-9H). The *res_clas* field groups imagery into standardized resolution levels, while *Polygon ID* indexes each individual footprint. Finally, the *Metadata URL* links directly to the USGS EarthExplorer page, which contains thumbnails and complete technical parameters, including film type, polarity, and four-corner geographic coordinates. After harmonization, downloaded footprint records from USGS EarthExplore were applied to generate *global_keyhole_free.gpkg* and *global_keyhole_full.gpkg*, each containing three layers (*Declass1*, *Declass2* and *Declass3*).

Since no attribute provides explicit numeric resolution values, an additional attribute was derived to classify images into standardized resolution classes. As the Keyhole images distributed by the United States Geological Survey are duplicates of the original films^30^, their effective resolution may be somewhat lower than the nominal specifications. Nevertheless, classification of resolution levels in this study followed documented technical specifications in the Declassified Intelligence Satellite Photographs Reports^2^. Accordingly, imagery with resolutions of ≤1 m (KH-7, KH-9H) was assigned to the meter-level class (C1), imagery with 1–3 m resolution (KH-4A, KH-4B and KH-6) to the three-meter class (C2), and imagery with 8–12 m resolution (KH-1, KH-2, KH-3, KH-4 and KH-9L) to the ten-meter class (C3) (Table 1). Extremely coarse imagery such as KH-5 (~140 m) was excluded. The free dataset includes 19,402 images in C1 (32.8%), 32,731 in C2 (55.4%), and 6,997 in C3 (11.8%). The full dataset includes 601,607 images in C1 (42.1%), 690526 images in C2 (48.3%), and 137,532 images in C3 (9.6%). Classification files (*classification_free.gpkg* and *classification_full.gpkg*) were provided, each comprising the *C1*, *C2*, *C3* and the combined layer *C0* layer. Since the cloud detection was not applied in this research, these images numbers could be interpreted as an upper bound on potentially usable imagery, particularly for the full dataset because users-ordered images are expected to avoid cloud cover.

**Table 1:** Resolution classification of Keyhole imagery missions.

Satellite	DataSets	Resolution (ft/m)	Start to End	Single Area (Km^2^)	Numbers (free/full)	Resolution classification
KH-7	D2	(2 to 4)/(0.9)	1963/7~1967/6	1,196 ± 980	1,462/16,474	C1
KH-9H	D3	(2 to 4)/(0.9)	1971/6~1984/10	6,789 ± 5,467	17,940/585,133	
KH-4B	D1	6/1.8	1967/9~1972/5	4,178 ± 982	13,990/185,569	C2
KH-4A	D1	9/2.8	1963/8~1969/9	5,749 ± 1,445	18,738/504,066	
KH-6	D1	6/1.8	1963/7~1963/8	Unknown	3/891	
KH-9L	D2	(20 to 30)/7.6	1973/3~1980/10	32,410 ± 7,704	4,349/28,888	C3
KH-3	D1	25/7.6	1961/8~1961/12	9,812 ± 1,352	510/9,740	
KH-4	D1	25/7.6	1962/2~1963/12	12,876 ± 9,611	1,848/90,583	
KH-2	D1	30/9.1	1960/12~1961/7	22,577 ± 15,832	238/7,059	
KH-1	D1	40/12.2	1960/8	9,670 ± 397	52/1,262	
KH-5	D1	460/153.3	1961/2~1964/8	337,664 ± 45,073	3,214/38,035	Not accounted

## Methods

### Global point grid generation

To establish a consistent spatial framework for evaluating the availability of Keyhole imagery, a global grid of points in the sinusoidal projection at a spatial resolution of 0.05° × 0.05° was applied as it provides an uninterrupted and equal area projection^31^. Totally 25 million points were generated corresponds to ~5.6 km in linear distance or ~30 km² in area, which is substantially smaller than the footprint of a typical Keyhole image. For comparison, single-scene coverage areas ranged from ~1,100 km² for KH-7, ~4,000–5,000 km^2^ for KH-4A/B, and up to ~32,000 km^2^ for KH-9L (Table 1). This scale relationship ensures that each image footprint typically intersects multiple grid points, thereby avoiding undersampling while retaining sufficient spatial detail to characterize coverage heterogeneity. Furthermore, the 0.05° resolution aligns with widely adopted standards in global environmental datasets as land cover^32^ and climate products^33^, allowing seamless integration of Keyhole-derived indicators into broader Earth system analyses.

### Image–Point intersection and indicator derivation

To quantify the spatiotemporal availability of Keyhole imagery, all image footprints were spatially intersected with the global 0.05° point grid. For each grid point that fell within one or more imagery polygons, a comprehensive set of indicators was derived to describe coverage frequency, temporal extent, data continuity, and resolution stability. These indicators provide a standardized basis for evaluating the suitability of locations for long-term geospatial analysis.

### Coverage frequency

Two metrics quantify the number of observations available at each point. *cover_count* records the total number of image scenes intersecting a point, while *unique_date_count* represents the number of distinct acquisition dates. For example, a point with *cover_count = 18* and *unique_date_count = 12* indicates that 18 images were acquired across 12 different dates, with multiple acquisitions occurring on certain days.

### Temporal extent

The observation history of each point was characterized by *first_observation_date* and *last_observation_date*, denoting the earliest and latest acquisitions, respectively. From these, *observation_time_span* was calculated as the elapsed time in years between the two endpoints. For instance, a point first observed on 1962-07-15 and last observed on 1982-11-03 yields an *observation_time_span* of 20.3 years.

### Peak observation density

To capture temporal clustering of imagery, *peak_year* and *peak_year_counts* identify the year with the maximum acquisitions (e.g., 1973 with five images). In addition, *peak_three_years* and *peak_three_years_counts* summarize the three-year interval with the densest coverage (e.g., 1972–1974 with eleven images).

### Scene detail

To ensure reproducibility and allow direct inspection of the underlying imagery, each covered point also includes a *scene_detail* attribute. This field stores a semicolon-delimited list of all intersecting scenes, with each entry containing the USGS metadata URL For instance, ‘https://earthexplorer.usgs.gov/scene/metadata/full/5e7c41f3ffaaf662/D3C1201-100004A040/; https://earthexplorer.usgs.gov/scene/metadata/full/5e7c41f3ffaaf662/D3C1201-100004F039/’.

### Temporal segmentation and coverage classification

To assess temporal heterogeneity, the full observation record (1960–1984) was divided into five standardized five-year intervals: T1 (1960-01 to 1964-12), T2 (1965-01 to 1969-12), T3 (1970-01 to 1974-12), T4 (1975-01 to 1979-12), and T5 (1980-01 to 1984-12). For each grid point, binary variables (1/0) indicate whether coverage exists within each interval. Combining these variables yields 32 unique temporal coverage classes (e.g., T1 only, T2 + T3, T1 + T3 + T4, etc.). While these classes provide maximum specificity, their number complicates visualization and interpretation. Therefore, we further aggregated them into five broader groups based on the number of intervals covered: (1) single-interval, (2) two-interval, (3) three-interval, (4) four-interval, and (5) full five-interval coverage. This two-level classification ensures both fine temporal specificity and practical usability for broader analyses.

### Resolution-coverage combinations

To characterize the interplay of imagery acquired at different spatial resolutions, we developed a classification scheme summarizing whether a point is covered by one or multiple resolution classes. This analysis was conducted on the C0_covered layer, which includes all points with at least one observation from any mission. Each covered point was assigned a categorical attribute *resolution_coverage* representing one of seven possible combinations in Table 2. This scheme enables rapid identification of regions with overlapping resolutions, which are particularly valuable for multi-resolution integration and cross-scale validation. By contrast, points in classes A–G represent areas constrained to a single resolution, which may limit fine-scale applications but still provide essential historical coverage. This ensures that both the conceptual framework and the quantitative proportions are clearly documented and accessible.

**Table 2:** Resolution-Coverage Classes (A–G) and Their Interpretation.

Class	Resolution-Coverage Definition	Interpretation and Relevance
A	Covered exclusively by meter-level imagery	Provides the finest detail available, supporting tasks such as building extraction, archaeological mapping, and fine-scale land-use studies.
B	Covered exclusively by three-meter imagery	Represents mid-level resolution coverage, suitable for regional agricultural monitoring, land-resource evaluation, and environmental assessments.
C	Covered exclusively by ten-meter imagery	Offers coarser but extensive coverage, supporting applications such as forest inventories, water-body mapping, and regional landscape analyses.
D	Covered jointly by meter- and three-meter imagery	Enables multi-scale validation and cross-resolution integration, improving reliability in land-use change detection.
E	Covered jointly by three- and ten-meter imagery	Provides consistent moderate-resolution coverage across large areas, useful for reconstructing long-term land-use dynamics.
F	Covered jointly by meter- and ten-meter imagery	Represents rare overlap of extreme resolutions, allowing both detailed and contextual perspectives in spatial analysis.
G	Covered by three resolution levels simultaneously	Represents the most data-rich areas in the archive, enabling comprehensive multi-resolution and multi-temporal analyses.

## Data Records

### Data file structure and storage format

The KRIST dataset^28^ uses GeoPackage (.gpkg) as intermediate storage throughout metadata harmonization, footprint classification and point-based statistical computation, and provides the final products as a paired netCDF (.nc) and HDF5 (h5) for efficient reuse. Specifically, *global_keyhole_free/full.gpkg* contains cleaned scene-level metadata, *classification_free/full.gpkg* provides reclassified footprint polygons. The final intersection and statistics products are distributed as a netCDF-HDF5 pair and are restricted to covered grids points only. The netCDF file stores numeric variables (e.g., *cover_count, unique_date_count*). The HDF5 file stores the complete list of metadata URLs for each grid point at that resolution, which could enable users to quickly check cloud conditions through thumbnails and locate download links within the selected area of interest. An overview of all files, their contents, and attributes is provided in Table 3.

**Table 3:** Overview of dataset files, layers, and key attributes.

File name	Layers/variables included	Description	Key attributes
global_keyhole_free.gpkg/global_keyhole_full.gpkg	Declass1, Declass2, Declass3	Raw metadata harmonized from USGS EarthExplorer for Declass 1–3 collections. Each layer corresponds to one dataset release stage.	All attributes same as downloaded shapefiles from USGS EarthExplorer
classification_free.gpkg/classification_full.gpkg	C1, C2, C3, C0	Resolution-specific imagery footprints (C1 = meter-level, C2 = three-meter-level, C3 = ten-meter-level). The combined layer C0 merges all three classes.	Entity ID, Acquisition, Mission, Satellite, Polygon ID, Metadata URL, Resolution Class (*res_clas*) fixed to a single value in C1–C3, and mixed in C0
global_grid.gpkg	global_grid	Global regular point grid at 0.05° resolution (~25 million points), serving as the sampling framework.	point_id, geometry (ESRI:54008)
free_C{0-3}_stas.nc fulll_C{0-3}_stas.nc	Numeric variables (point-based indicators)	Point-level intersection results between the global grid and imagery footprints. Only covered grid points are included.	point_id, cover_count, unique_date_count, first_observation_date, last_observation_date, observation_time_span, peak_year, peak_year_counts, peak_three_years, peak_three_years_counts, temporal_coverage
free_C{0–3}_scene_detail.h5 / full_C{0–3}_scene_detail.h5	Per-point metadata URL lists	The complete list of metadata URLs for each covered grid point and resolution.	Point_id, list of metadata URLs

### Attribute definitions

Two groups of attributes are defined within the dataset, reflecting the dual structure of the data. The first group (Table 4) corresponds to the imagery footprint metadata stored in *classification_free/full.gpkg*. These attributes describe the basic characteristics of each Keyhole scene and provide direct links to the USGS EarthExplorer metadata pages. The second group (Table 5) corresponds to the point-based spatiotemporal indicators distributed as resolution-specific netCDF files (free/full_C{0-3}_stats.nc) for covered grid points, and per-point metadata URLs restored in paired HDF5 files (free/full_C{0-3}_scene_detail.h5). Together, these two sets of attributes provide complementary perspectives: the first describes the imagery at the scene level, while the second characterizes long-term coverage properties at regular spatial intervals.

**Table 4:** Attributes of imagery footprint layers (from *classification_free/full.gpkg*).

Field	Definition	Example Value
Entity ID	Unique identifier of the image scene	D3C1201-100004A017
Acquisition	Acquisition date (ISO 8601 format)	1971/6/16
Mission	Mission code identifying the operational cycle	1201-1
Satellite	Satellite series designation	KH-9H
res_clas	Standardized resolution class (C1, C2, C3)	C1
Polygon ID	Unique index for each footprint polygon	1418
Metadata URL	Link to full USGS metadata and thumbnail preview	https://earthexplorer.usgs.gov/scene/metadata/full/5e7c41f3ffaaf662/D3C1201-100004A017/

**Table 5:** Point-based spatiotemporal attributes (from *free/full_C{0-3}_stats.nc and free/full_C{0-3}_scene_detail.h5*).

Field name	Type	Unit	Description
point_id	Integer	—	Unique identifier for each grid point
cover_count	Integer	count	Total number of image footprints covering the point
unique_date_count	Integer	count	Number of unique observation dates
first_observation_date	Date	YYYY-MM-DD	Date of the earliest image observation
last_observation_date	Date	YYYY-MM-DD	Date of the latest image observation
observation_time_span	Float	years	Time span between first and last observation
peak_year	Integer	year	Year with the highest number of observations
peak_year_counts	Integer	count	Number of observations in the peak year
peak_three_years	String	years	Three-year window with the most observations (e.g., 1972–1974)
peak_three_years_counts	Integer	count	Number of observations within the peak three-year period
T1–T5	Binary	0/1	Whether the point was observed in each of the five 5-year intervals
temporal_coverage	String	—	Five-year-period combination (e.g., T2/T3/T5)
resolution_coverage	Categorical	A–G	Resolution composition for C1–C3 coverage (only for C0-covered points)
scene_detail	String	—	Concatenated list of metadata for all intersecting images

### Data quality and compliance

All temporal fields were normalized to the ISO 8601 date format. Spatial coordinates were expressed in the sinusoidal projection (ESRI:54008), guaranteeing compatibility with widely used geospatial datasets and tools. Logical validation was performed to ensure consistency across records (e.g., observation spans), and duplicate or incomplete records were removed during preprocessing. By combining rigorous harmonization, standardized attribute definitions, and open-standard storage, the dataset provides a reliable foundation for global analyses of Keyhole imagery.

## Technical Validation

To ensure the reliability and usability of the dataset, we conducted a series of validation analyses focusing on spatial coverage, temporal completeness, and resolution diversity. These evaluations are intended to demonstrate that the dataset provides both consistent global coverage and meaningful temporal sampling, thereby supporting subsequent applications in land use, environmental change, and historical landscape reconstruction. The KRIST dataset includes both the free and full achieve of Keyhole imagery, and we used parts of the free dataset as an example to briefly describe the spatial-temporal distribution of the Keyhole imagery. Detailed figures showing the resolution-specific imagery for free and full datasets were provided in the Supplementary Information.

### Temporal validity of free dataset

The free dataset could be outdated as the number of free downloaded images increases over time as people purchase the non-free downloaded images. To demonstrate the temporal evolution of the free dataset of KRIST, we obtained the number of the free images at two time points of June 20, 2025 and November 25, 2025 (Table 6). The counts of free images for each Declass collection increased over this half year. Users could re-download the latest free Keyhole collection and apply the analysis code provided in this work to obtain the up-to-date spatial-temporal distribution of free imagery.

**Table 6:** Image count of free Keyhole at 2025/6/20 and 2025/11/25.

	Image count		Percentage increase (%)
	2025/6/20	2025/11/25	
Declass1	38,769	38,939	0.4
Declass2	5,705	5,820	2.0
Declass3	17,522	17,940	2.4

### Global spatial coverage overview

**Fig. 1:**
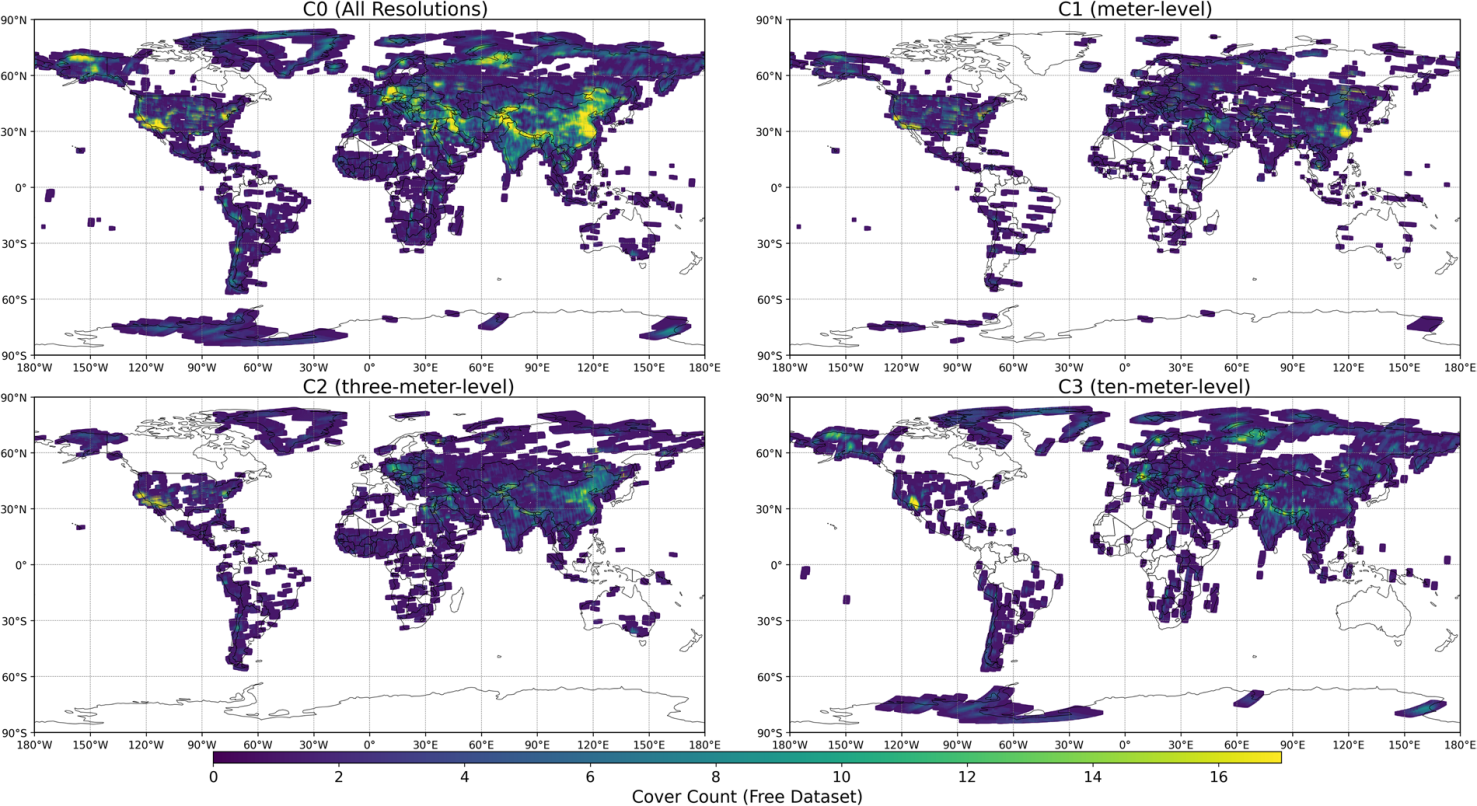
Distribution of cover counts for different resolution levels of free dataset: (**a**) C0 (all resolutions), (**b**) C1 (meter-level), (**c**) C2 (three-meter-level), and (**d**) C3 (ten-meter-level).

To validate the spatial and temporal robustness of the dataset, we first analyzed the distribution of observation frequencies (*cover count*) for each resolution level (Fig. 1 for free dataset and Supplement Fig. S1 for full dataset). For visual clarity all figures following were presented in the WGS 1984, although the dataset created in this work were in sinusoidal projection. The median number of observations was 3 for C0 (all resolutions combined), 2 for C1–C3. More than 70% of the grid points had no more than five scenes (72.8% for C0, 85.1% for C1, 87.4% for C2, and 86.1% for C3). In contrast, only a small portion of points were observed intensively, with more than 15 scenes available for 5.6% of C0 points, but less than 3% for C1–C3. Regions with higher coverage are primarily located in western and southern Europe, northeastern Russia, and southeastern Asia including China and India, as well as North America, while large areas of Canada, South America, southern Africa, and Australia lack imagery coverage. These results indicate that although the archive provides extensive coverage, the spatial density of observations is highly uneven, and only certain hotspots exhibit dense temporal sampling.

### Temporal span distribution

**Fig. 2:**
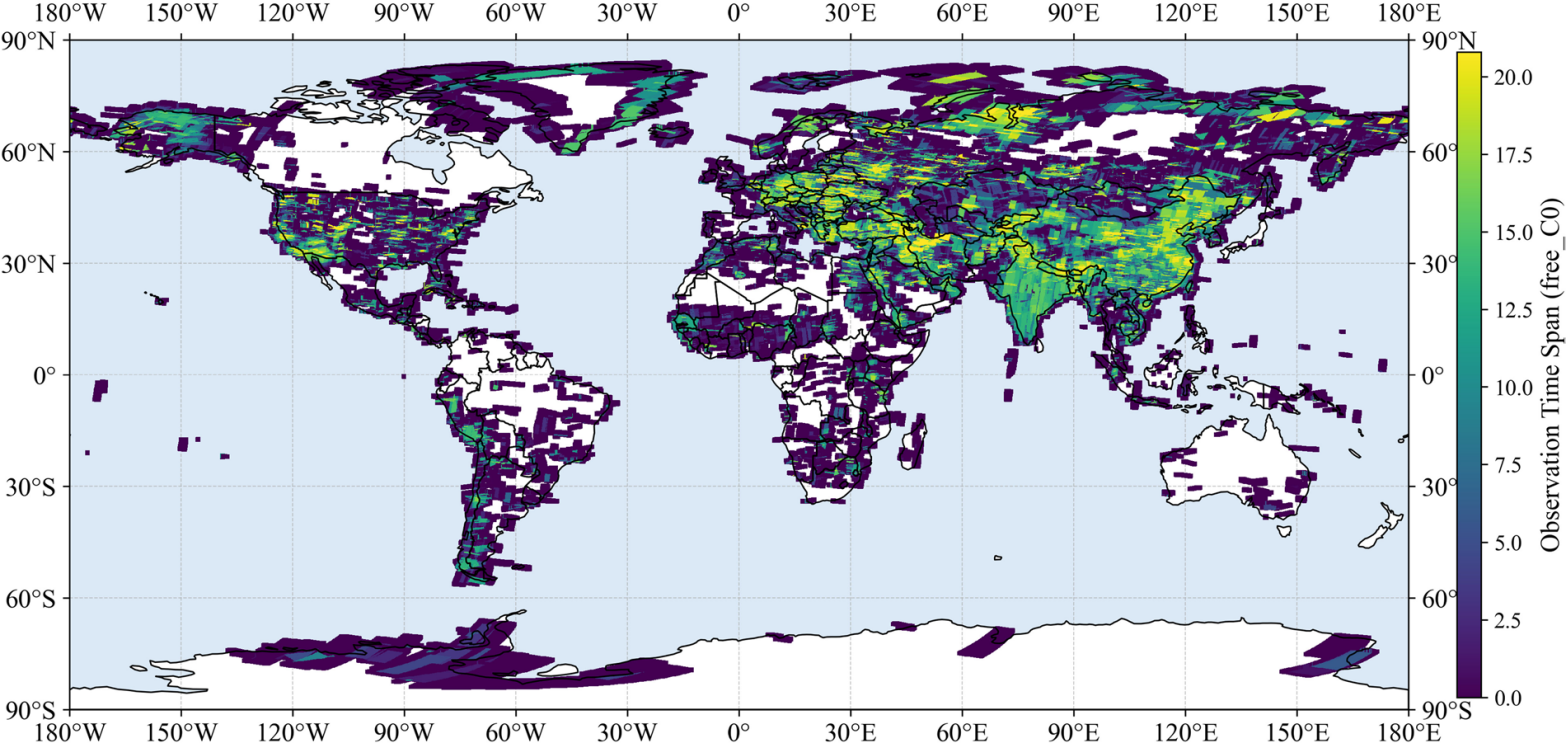
Distribution of temporal coverage span (first–last year difference) for C0 (all resolutions) of free dataset.

We further assessed the temporal span of observations by calculating the difference between the first and last acquisition year for each grid point. For C0 of free dataset the average span was 5.8 years, while the median was only 2.1 years (Fig. 2). The interquartile range (IQR) extended from 0 to 11.9 years, indicating that although many locations have very limited temporal coverage, a subset experienced a decade or more of repeated acquisitions. More than half of the points (57.9%) had spans shorter than five years, and only 14.4% of points exhibited spans longer than 15 years. The spatial distribution of temporal spans closely mirrors that of cover counts, with longer spans concentrated in regions where observation frequencies are also higher. These results suggest that while the archive provides valuable snapshots across multiple periods, long-term continuous records are restricted to a smaller portion of the globe. Resolution-specific results (C1–C3) of free dataset exhibit similar distributional patterns, with short spans dominating and only a limited fraction of points showing spans longer than 15 years (Figs. S2–4). Temporal span distributions of full dataset for different resolutions were also provided (Figs. S5–8).

### Peak year distribution

**Fig. 3:**
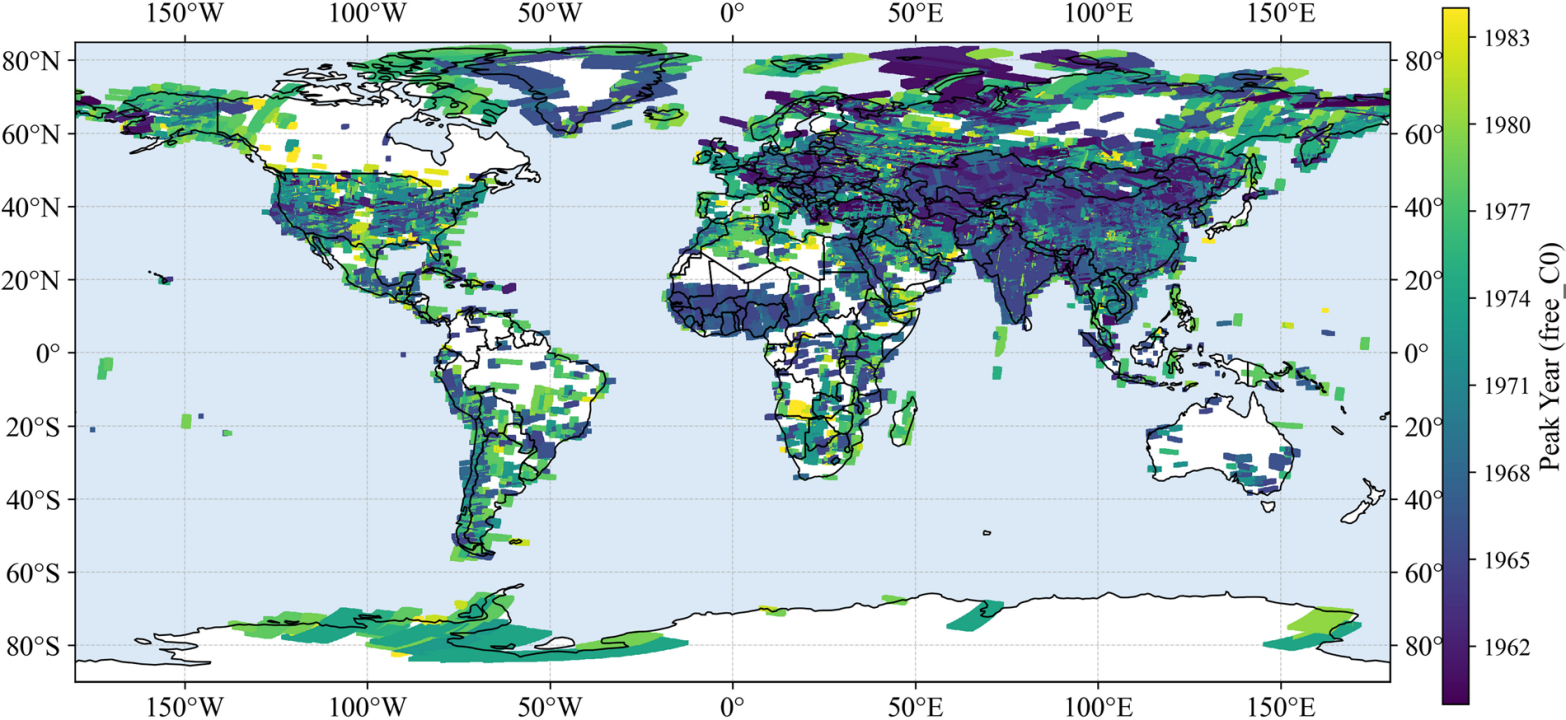
Distribution of peak years across grid points for C0 (all resolutions) of free dataset.

To characterize when the densest observations occurred, we identified the *peak year* for each grid point, defined as the year with the highest number of acquisitions within a ± 2-year window. The distribution for C0 reveals a strong temporal concentration in the mid-1960s and early-to-mid 1970s (Fig. 3). The most prominent peaks were in 1965 (12.6%), 1966 (5.4%), and 1967 (6.0%), followed by another cluster in 1973–1974, accounting for 6.3% and 7.3% of points, respectively. Smaller peaks were observed around 1962 (8.1%) and 1978 (5.2%). In contrast, very few points had peak years in the early 1960s or after 1980, with percentages generally below 1%. This distribution confirms that the archive is not evenly spread across time but is instead dominated by two major observation phases: the mid-1960s and the early-to-mid 1970s. These phases align with the historical deployment of successive Keyhole missions and the expansion of reconnaissance coverage during the late Cold War period. Resolution-specific results (C1–C3) show consistent peak-year clustering and are provided in the Supplementary Information (Figs. S9–11). Peak year distributions of full dataset for different resolutions were also provided (Figs. S12–15).

### Resolution coverage combinations

**Fig. 4:**
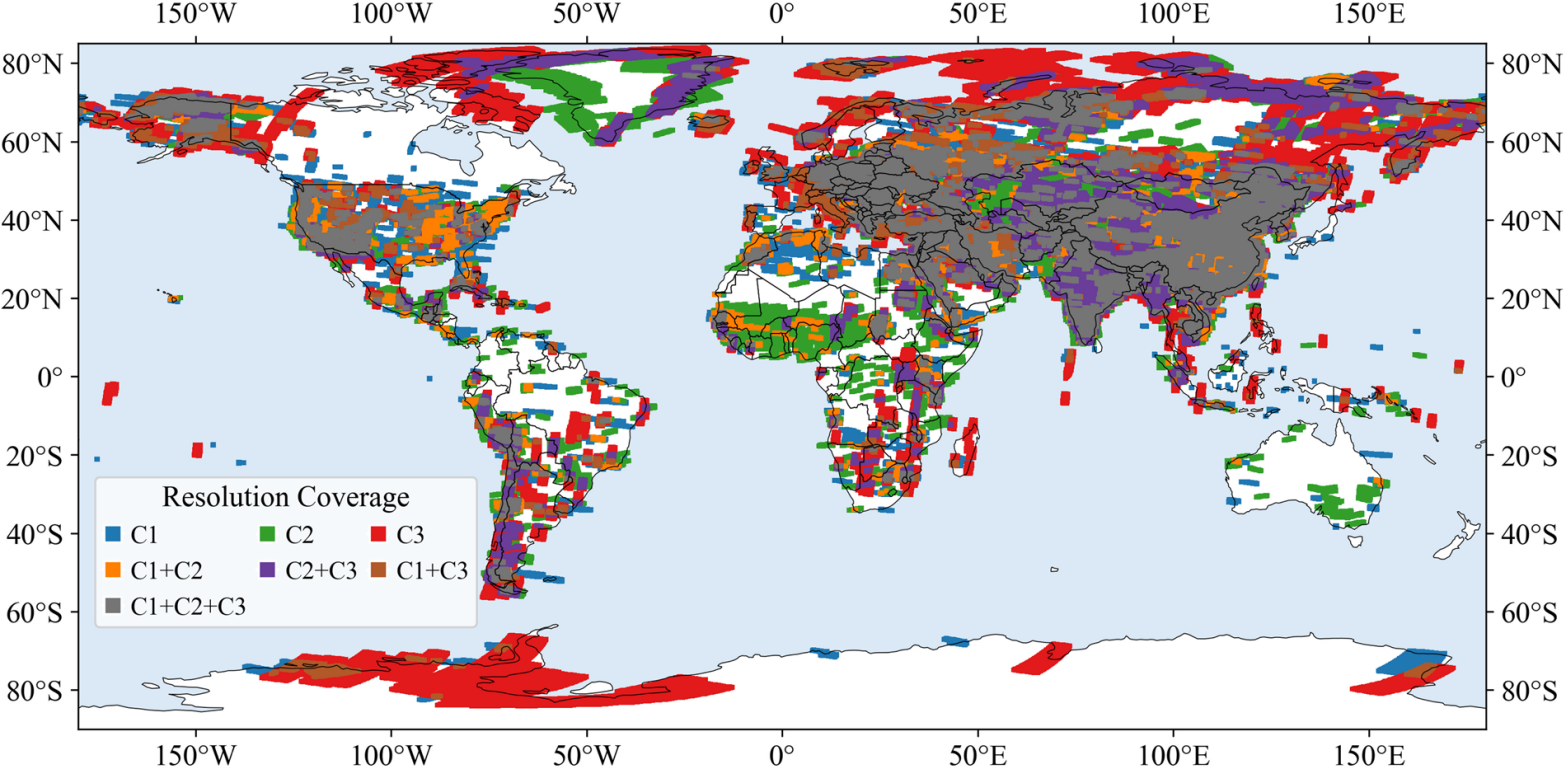
Distribution of resolution coverage combinations (**A–G**) across grid points of free dataset.

We also examined the distribution of resolution combinations to evaluate the multi-scale potential of the dataset. Each grid point was classified according to the availability of imagery from different resolution levels (C1, C2, C3), with seven possible classes (A–G) (Fig. 4). The results show that single-resolution coverage is most common: 34.5% of points were covered exclusively by C3 (class C), 16.2% by C2 (class B), and 8.1% by C1 (class A). In contrast, multi-resolution coverage is also significant. About 16.2% of points fell into class E (C2 + C3), 6.3% into class F (C1 + C3), and 5.2% into class D (C1 + C2). Notably, 13.5% of points (class G) were covered by all three resolution levels simultaneously. This distribution indicates that while many regions are represented by only one resolution, a substantial proportion of the globe benefits from overlapping coverage at multiple resolutions. Such diversity ensures that the dataset can support applications across different spatial scales, ranging from fine-grained local analysis to broader regional studies. Resolution coverage combinations distributions of full dataset were also provided (Fig. S16).

### Temporal coverage combinations

**Fig. 5:**
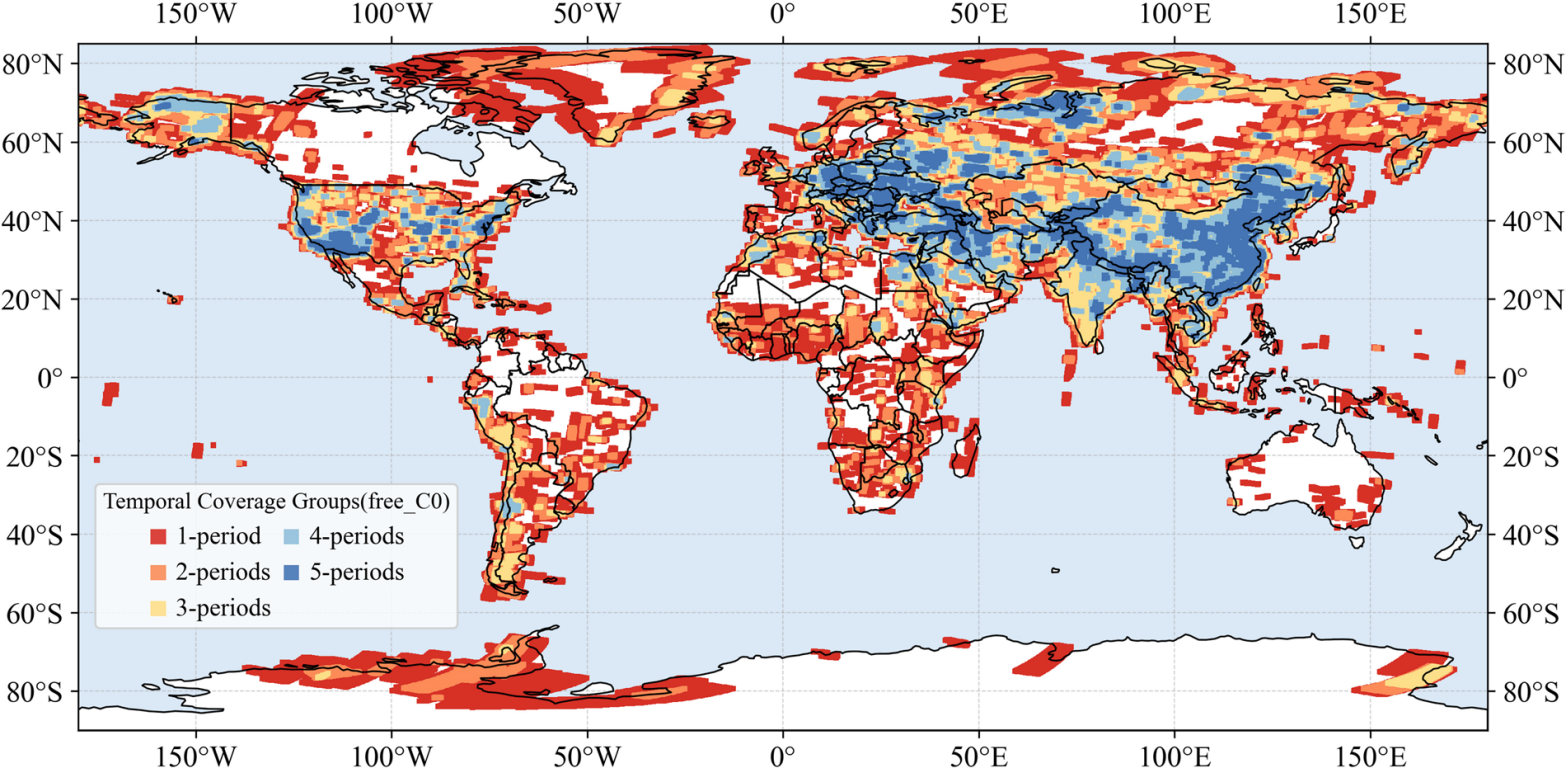
Distribution of temporal coverage combinations (T1–T5) for C0 of free dataset.

To assess the multi-period potential of the dataset, we classified each grid point according to its coverage across the five defined time periods (T1: 1960–1964; T2: 1965–1969; T3: 1970–1974; T4: 1975–1979; T5: 1980–1984). For C0, a total of 31 unique temporal-coverage combinations were identified, reflecting the diverse ways in which different regions were repeatedly imaged. The most common class was coverage in both T2 and T3, accounting for 18.4% of all points (Fig. 5). Other frequent categories included T2-only (12.3%), T4-only (9.8%), and T3 + T4 + T5 (5.5%). In contrast, full five-period coverage was rare, observed in only 0.3% of points. Areas with multi-period coverage (more than four periods) are primarily concentrated in eastern and western China, the Middle East, western Europe, and northeastern Russia. More than half of the grid points fell into combinations involving two or more time periods, indicating that repeated observations across different decades were widespread, although continuous monitoring across the full 25-year span was very limited.

This distribution demonstrates that the dataset has strong potential for multi-period studies of land use and environmental change, particularly when focusing on transitions between adjacent periods (e.g., T2–T3 or T3–T4), but it also highlights the scarcity of long-term continuous records across all five periods. Resolution-specific results (C1–C3) show comparable patterns, with most points covered in only one or two periods and very few achieving full five-period coverage (Figs. S17–19). Temporal coverage combination distributions of full dataset for different resolutions were also provided (Figs. S20–23).

### Overall assessment

The validation analyses presented above provide a comprehensive overview of the spatial, temporal, and resolution characteristics of the dataset. Coverage frequency analysis demonstrated that while most locations were observed only a few times, a subset of points received dense temporal sampling. Temporal span showed that the dataset captures repeated observations across decades, but long-term continuous coverage is limited to relatively few locations. The distribution of peak yeSars further revealed two major phases of intensive acquisitions, in the mid-1960s and early-to-mid 1970s. Resolution and temporal coverage combinations highlighted the dataset’s diversity: many regions are covered at multiple resolutions, and more than half of the points contain imagery from two or more distinct time periods. These features ensure the applicability of the dataset to multi-scale and multi-temporal studies. At the same time, the heterogeneity in coverage frequency, and temporal span underscores the need for careful consideration when applying the data to long-term monitoring or quantitative change detection. Taken together, these validations confirm that the dataset provides robust global coverage with strong potential for historical land use, environmental, and landscape research, while also documenting its inherent limitations in temporal continuity.

## Usage Notes

This dataset KRIST is designed to support a broad range of Keyhole applications in land use, environmental change, and historical landscape research. The global spatial coverage and multi-period acquisitions provide valuable baselines for investigating land cover prior to the era of modern remote sensing. In particular, the data can be used to (i) reconstruct historical land use patterns at regional to global scales, (ii) evaluate landscape change trajectories between the 1960s and 1980s, and (iii) provide reference information for studies of soil conservation, agricultural expansion, urbanization, and ecological restoration.

When applying the dataset, users should be aware of its heterogeneity and temporal validity. Cloud distribution or percentage were not accounted in this release, so the reported cover count might exceed the number of images that are practically interpretable for surface analysis. Using the metadata URLs included in the dataset, users can inspect the thumbnails for their region of interest to select images with acceptable cloud conditions. As demonstrated in the validation analyses, coverage frequency and temporal span vary considerably among locations, with some points exhibiting only one or two observations, while others provide dense coverage across multiple decades. Researchers are therefore encouraged to carefully select study areas and periods according to their research objectives, and to combine the dataset with complementary sources such as Landsat or regional aerial photography where finer temporal continuity is required^34^. The diversity of resolution combinations (meter-, three-meter-, and ten-meter-level imagery) also enables multi-scale investigations. Applications may range from fine-grained local analyses (e.g., settlement expansion, gully development) to broad-scale studies (e.g., agricultural land change, deforestation, or infrastructure development). Users should consider the trade-offs between spatial detail and temporal completeness when designing their studies.

Overall, this dataset provides a unique and robust foundation for extending land use and environmental change research into the pre-Landsat era. While it cannot fully replace modern satellite archives in terms of temporal continuity, it serves as an essential historical reference, bridging a critical gap in long-term landscape monitoring and offering new opportunities to integrate past and present Earth observation data.

## Supplementary information

Supplementary information

## Data Availability

The data are available on Figshare (10.6084/m9.figshare.30017944)^28^.

## Code availability

The processing codes are available on Figshare (10.6084/m9.figshare.30017944)^28^.
