# Supplementary material for: Global 0.05° Grid-Based Dataset of Keyhole Imagery with Spatio-Temporal Indicators (1960–1984)
Source: Sci Data. 2026 Feb 17;13:463. doi: 10.1038/s41597-026-06866-4 (PMC13021963; doi:10.1038/s41597-026-06866-4)
Supplement: Supplementary file 1 — Supplementary information [file 41597_2026_6866_MOESM1_ESM.pdf]

Supplement of "Global 0.1° Grid-Based Dataset of Keyhole Imagery with Spatio-Temporal Indicators (1960–1984)"

December 16, 2025

List of Figures:

S1 Distribution of cover counts for different resolution levels of full dataset 2
S2 Distribution of temporal coverage span for C1 of free dataset 2
S3 Distribution of temporal coverage span for C2 of free dataset 3
S4 Distribution of temporal coverage span for C3 of free dataset 3
S5 Distribution of temporal coverage span for C0 of full dataset 4
S6 Distribution of temporal coverage span for C1 of full dataset 4
S7 Distribution of temporal coverage span for C2 of full dataset 5
S8 Distribution of temporal coverage span for C3 of full dataset 5
S9 Distribution of peak year for C1 of free dataset 6
S10 Distribution of peak year for C2 of free dataset 6
S11 Distribution of peak year for C3 of free dataset 7
S12 Distribution of peak year for C0 of full dataset 7
S13 Distribution of peak year for C1 of full dataset 8
S14 Distribution of peak year for C2 of full dataset 8
S15 Distribution of peak year for C3 of full dataset 9
S16 Distribution of resolution coverage of full dataset 9
S17 Distribution of temporal coverage for C1 of free dataset 10
S18 Distribution of temporal coverage for C2 of free dataset 10
S19 Distribution of temporal coverage for C3 of free dataset 11
S20 Distribution of temporal coverage for C0 of full dataset 11
S21 Distribution of temporal coverage for C1 of full dataset 12
S22 Distribution of temporal coverage for C2 of full dataset 12
S23 Distribution of temporal coverage for C3 of full dataset 13

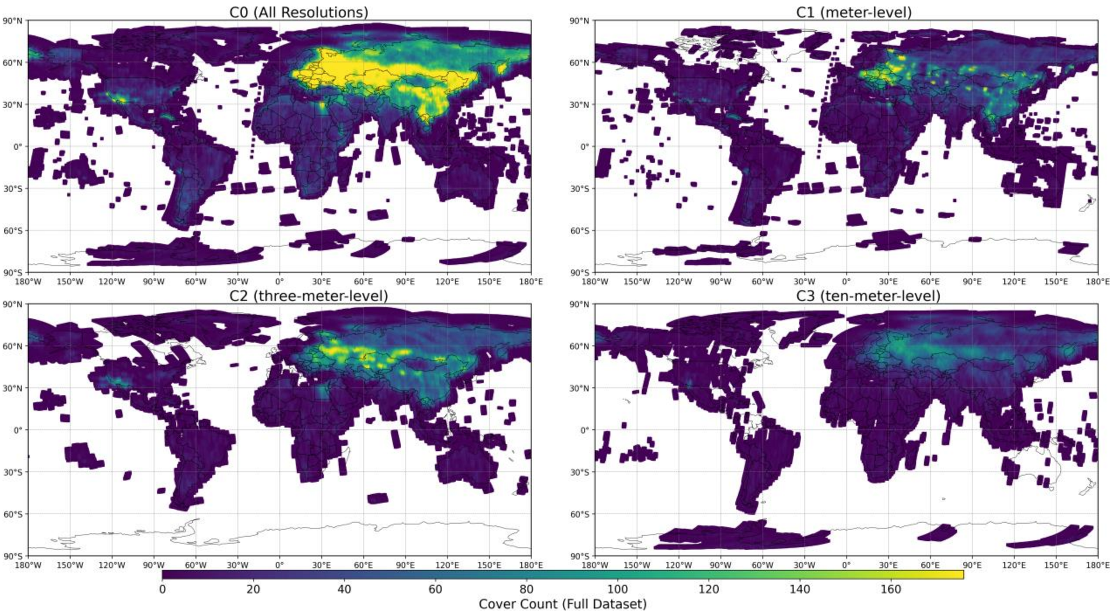

Figure S1. Distribution of cover counts for different resolution levels of full dataset: (a) C0 (all resolutions), (b) C1 (meter-level), (c) C2 (three-meter-level), and (d) C3 (ten-meter-level).

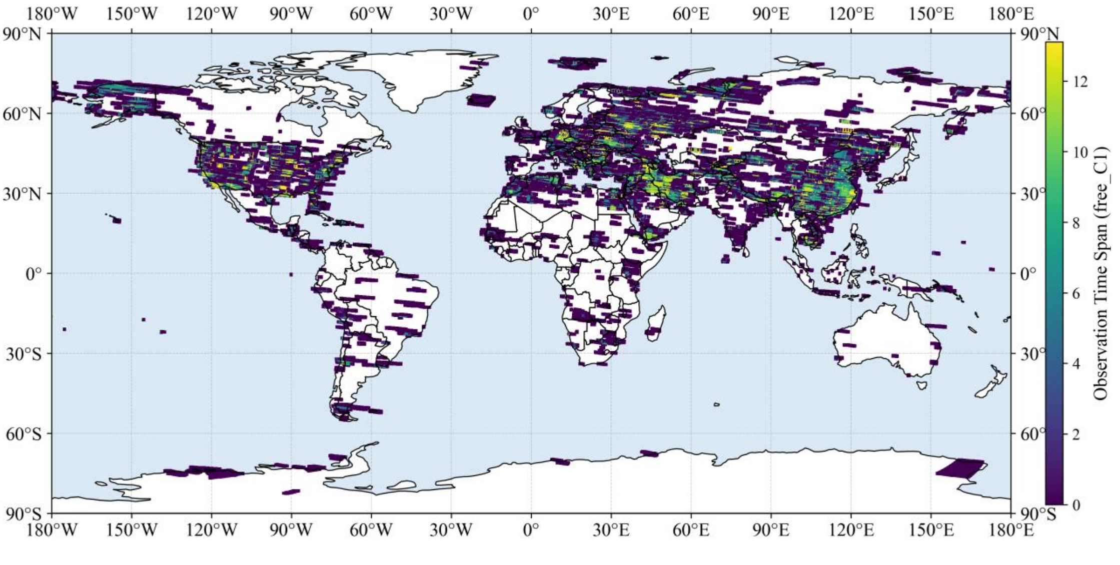

Figure S2. Distribution of temporal coverage span for C1 of free dataset

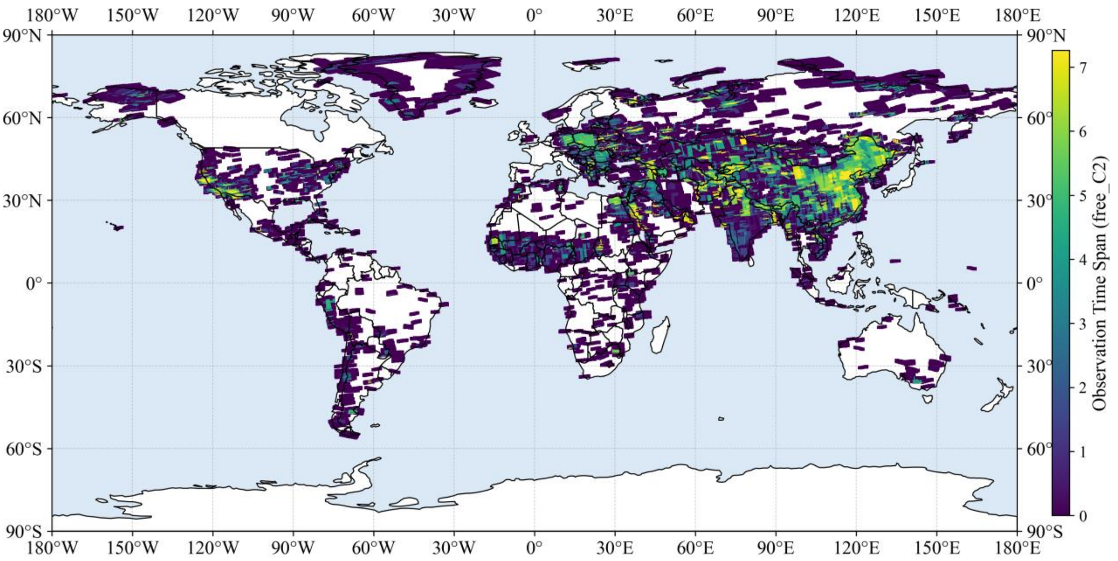

Figure S3. Distribution of temporal coverage span for C2 of free dataset

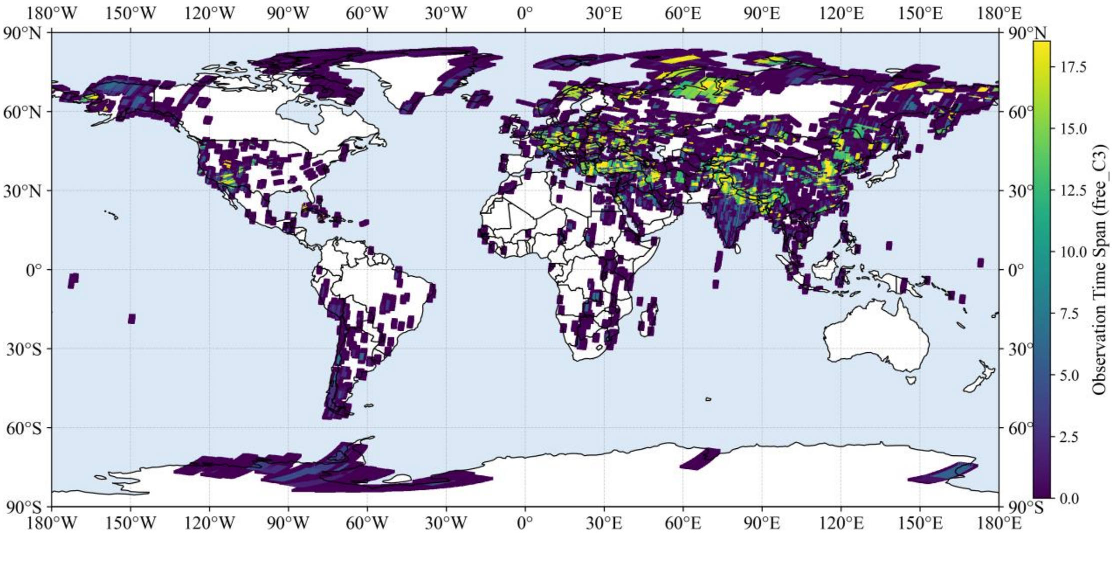

Figure S4. Distribution of temporal coverage span for C3 of free dataset

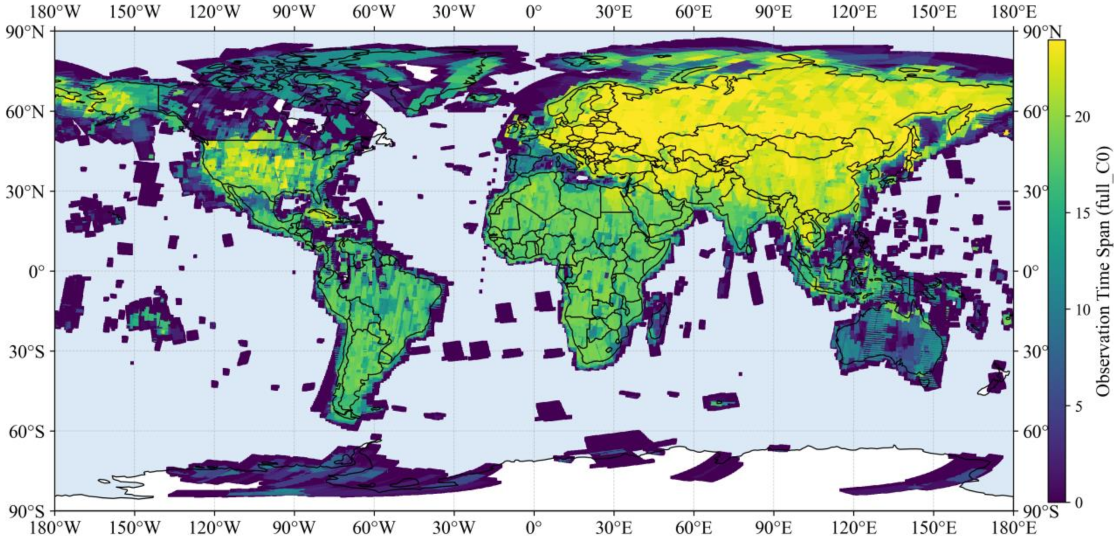

Figure S5. Distribution of temporal coverage span for C0 of full dataset

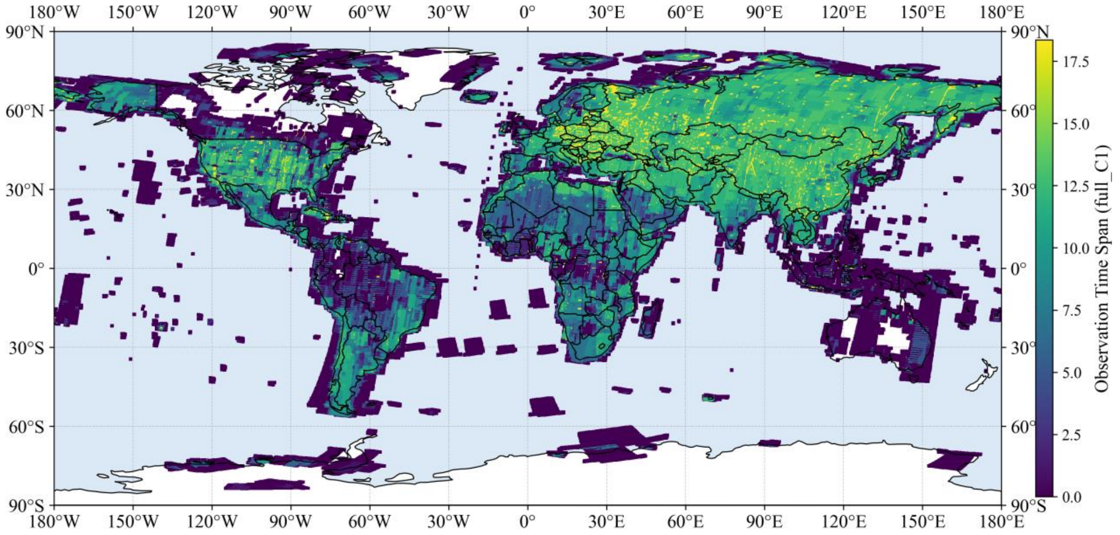

Figure S6. Distribution of temporal coverage span for C1 of full dataset

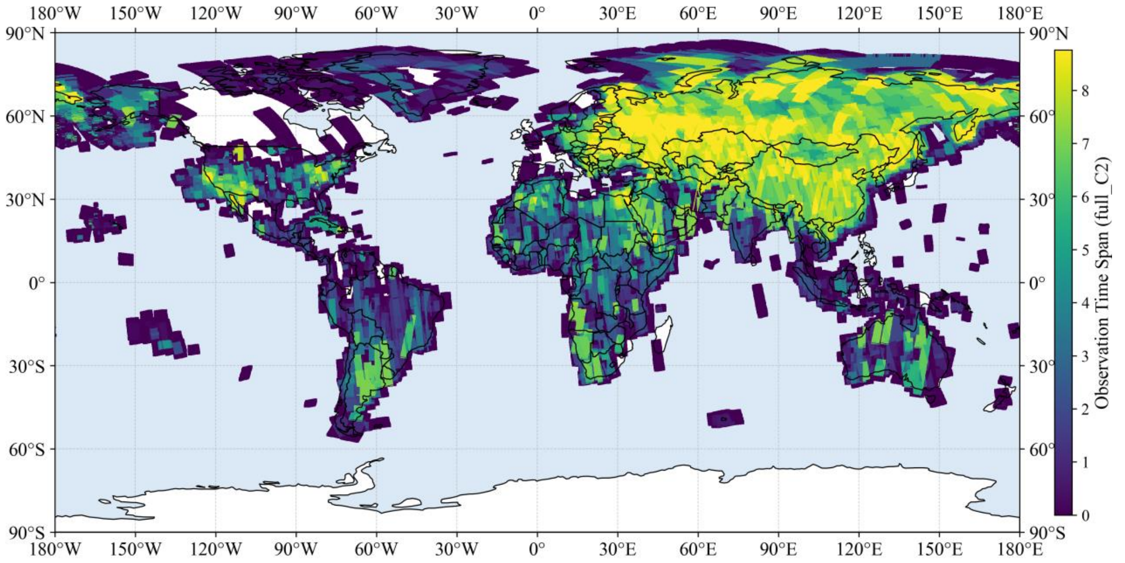

Figure S7. Distribution of temporal coverage span for C2 of full dataset

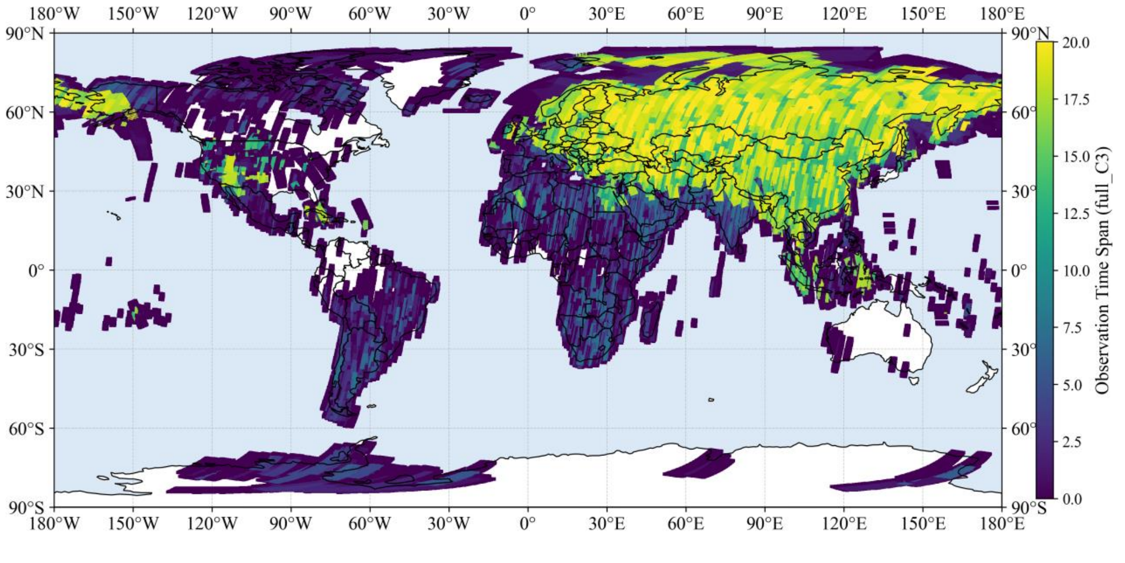

Figure S8. Distribution of temporal coverage span for C3 of full dataset

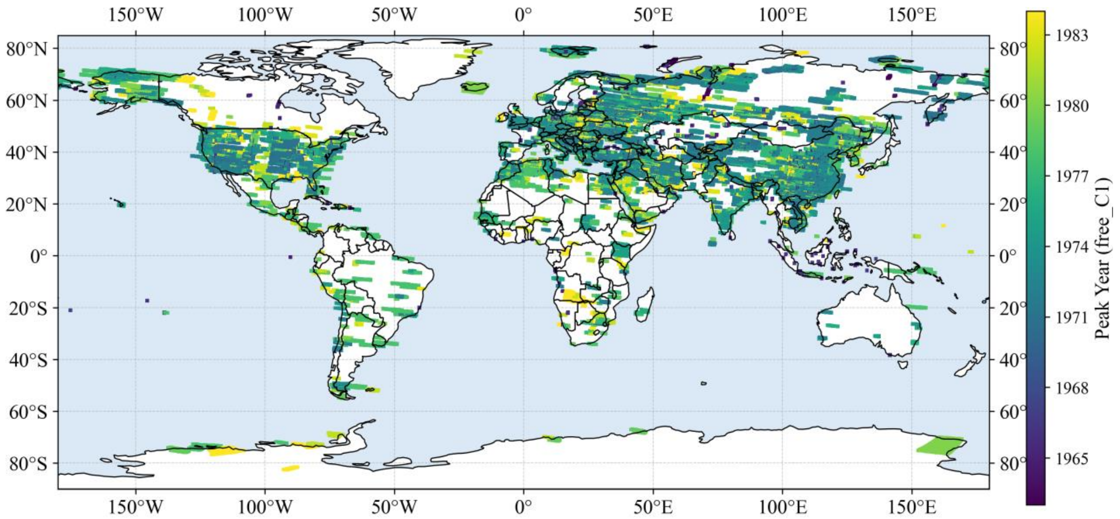

Figure S9. Distribution of peak year for C1 of free dataset

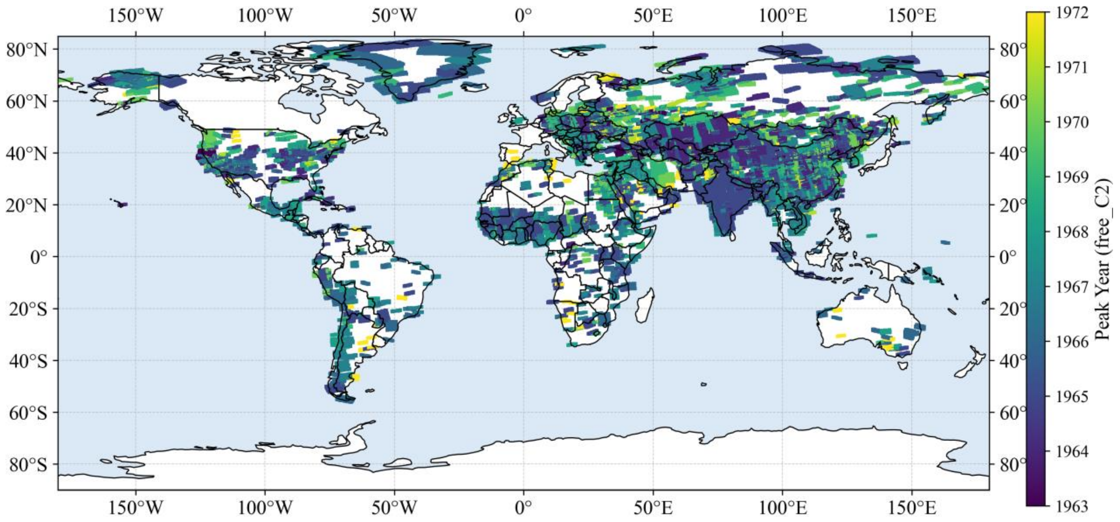

Figure S10. Distribution of peak year for C2 of free dataset

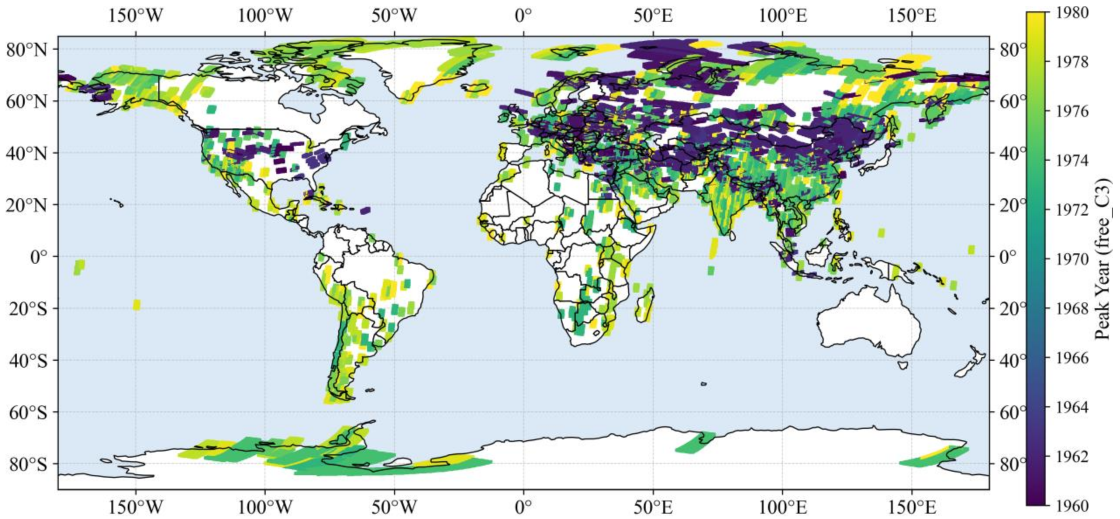

Figure S11. Distribution of peak year for C3 of free dataset

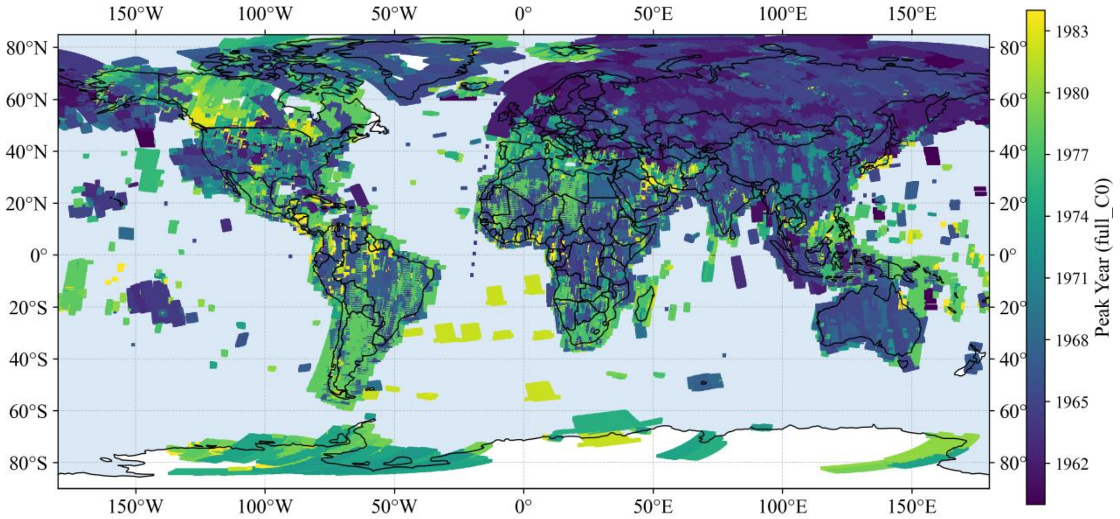

Figure S12. Distribution of peak year for C0 of full dataset

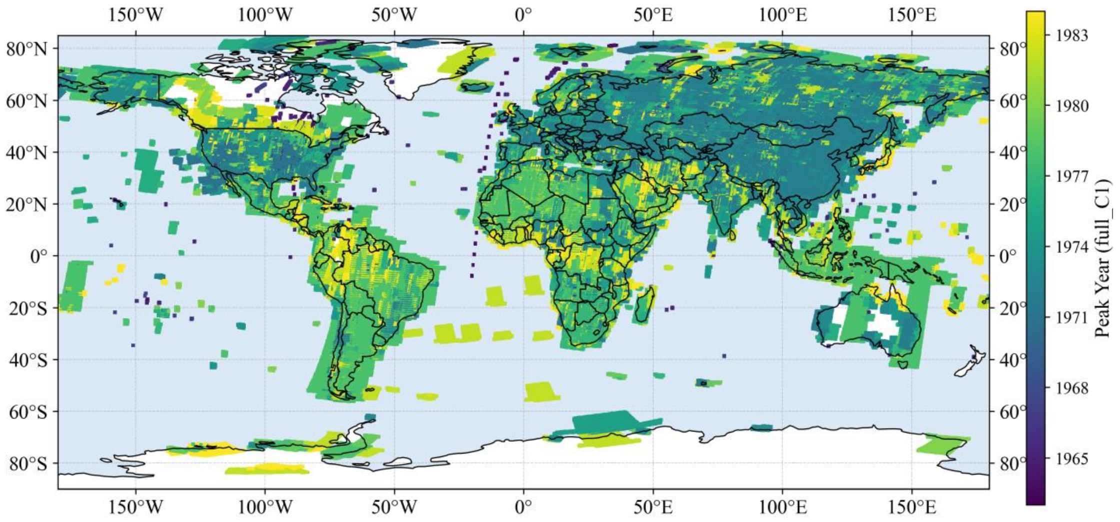

Figure S13. Distribution of peak year for C1 of full dataset

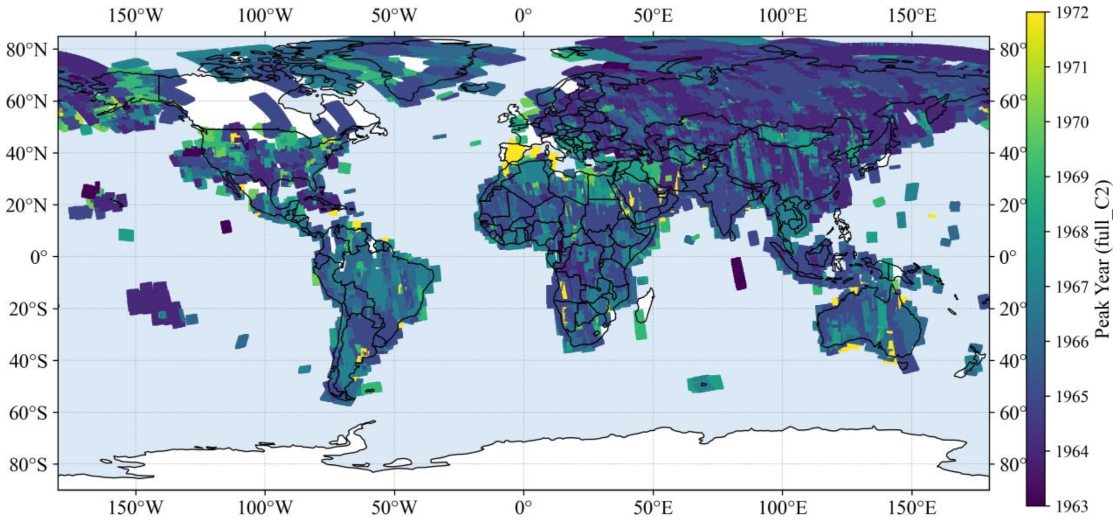

Figure S14. Distribution of peak year for C2 of full dataset

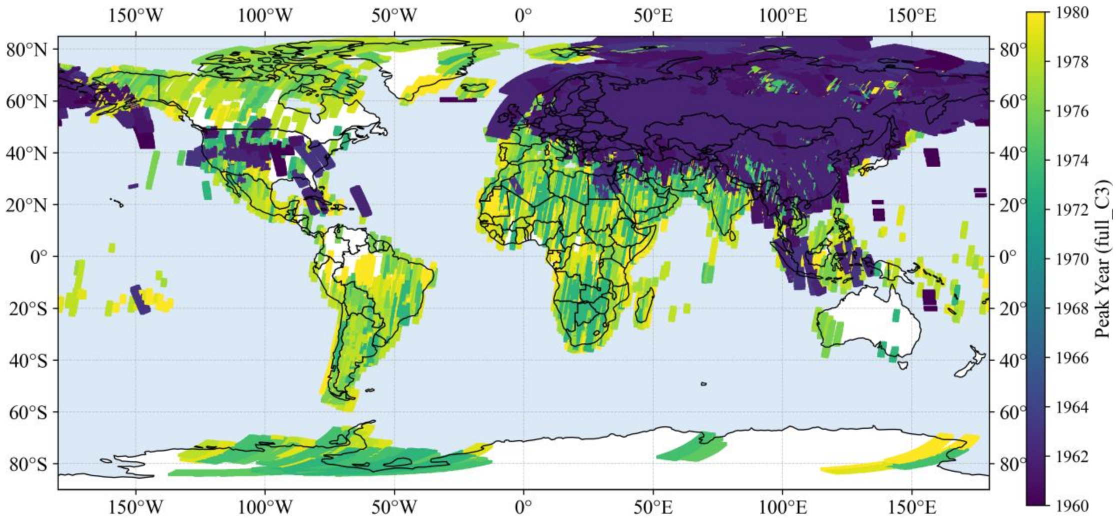

Figure S15. Distribution of peak year for C3 of full dataset

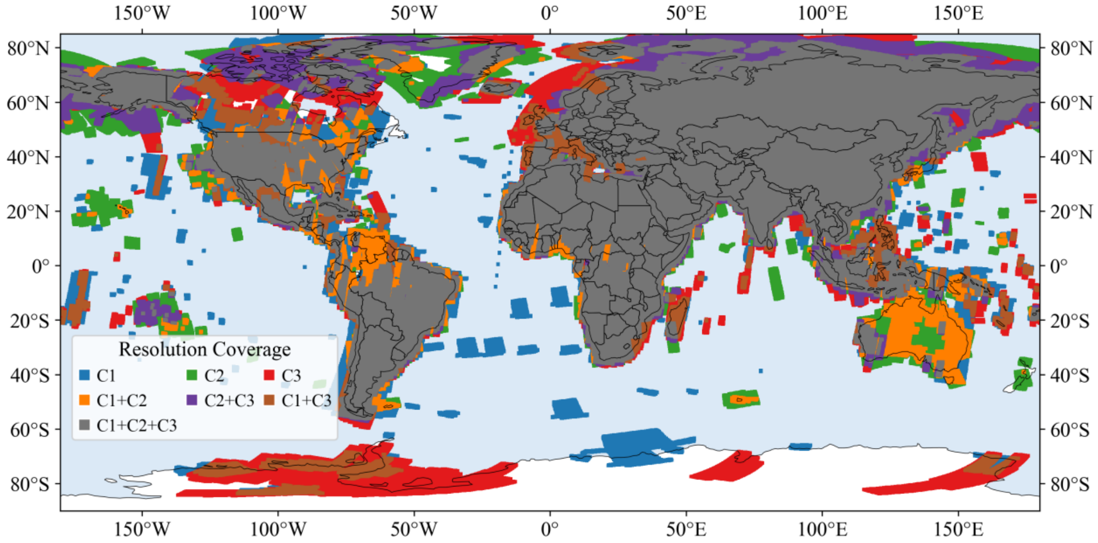

Figure S16. Distribution of resolution coverage of full dataset

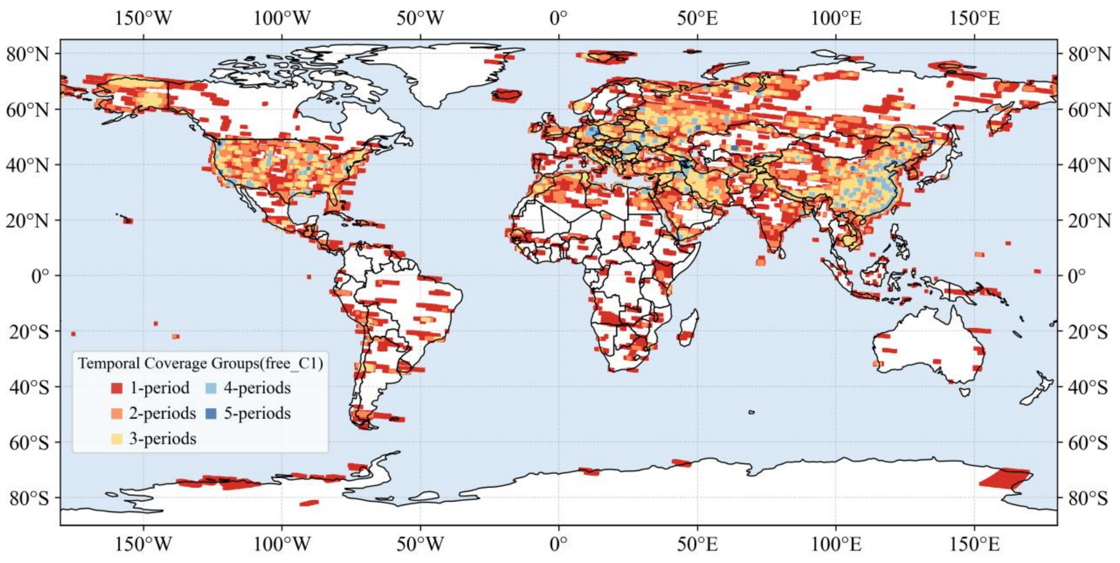

Figure S17. Distribution of temporal coverage for C1 of free dataset

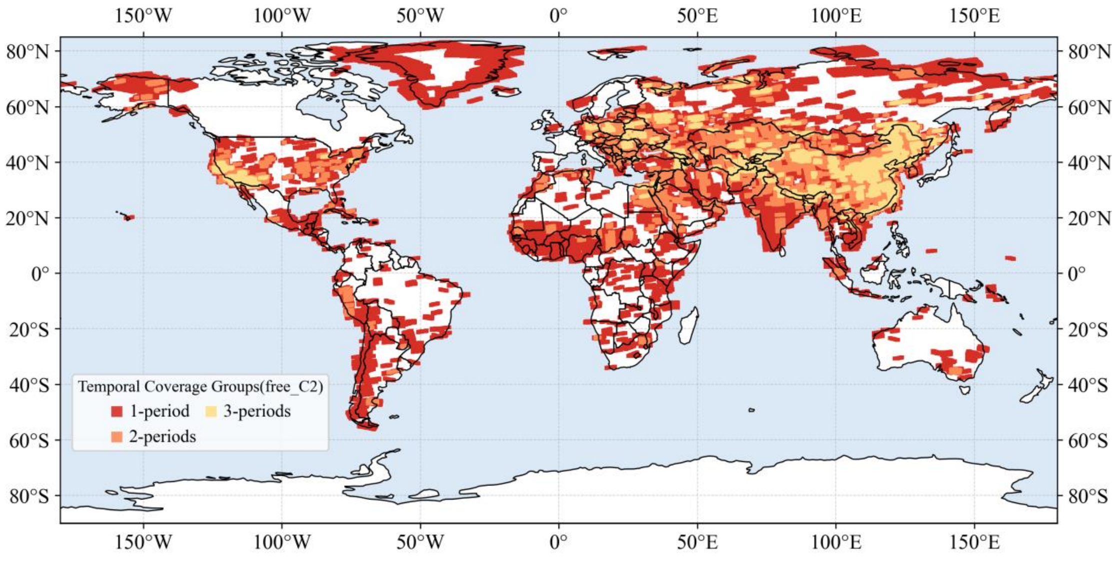

Figure S18. Distribution of temporal coverage for C2 of free dataset

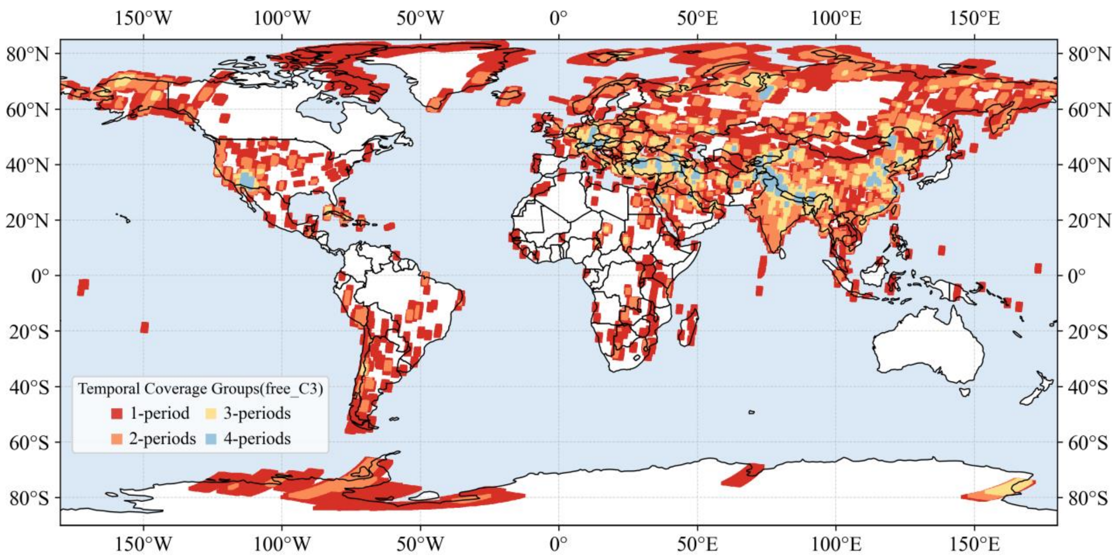

Figure S19. Distribution of temporal coverage for C3 of free dataset

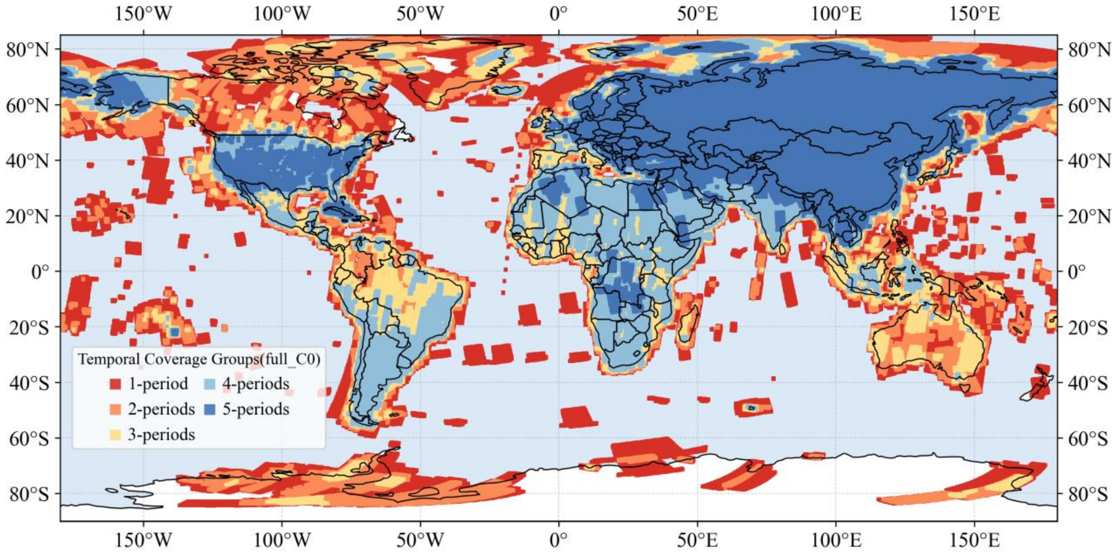

Figure S20. Distribution of temporal coverage for C0 of full dataset

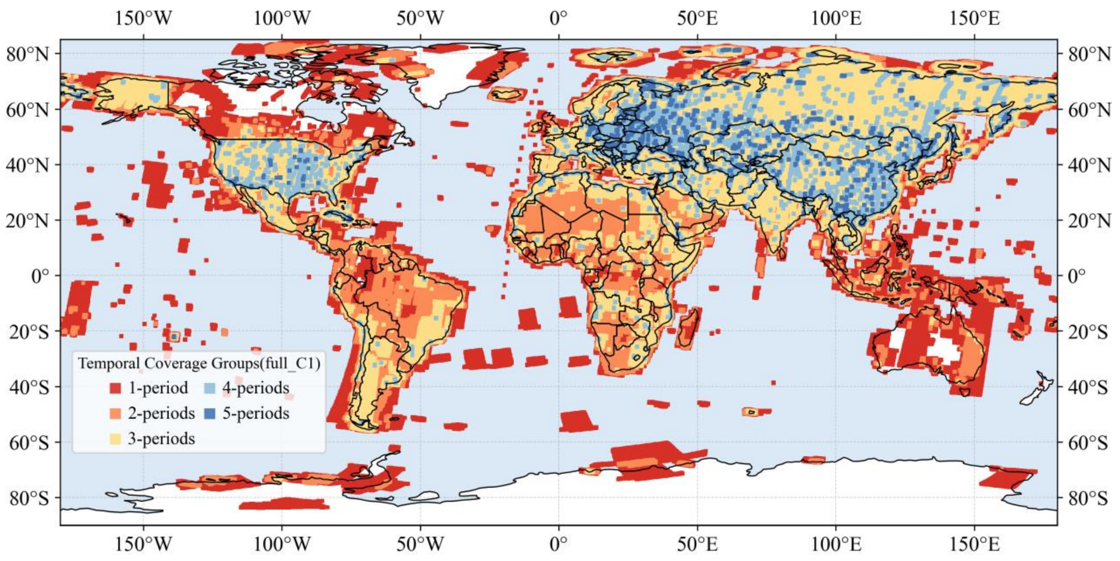

Figure S21. Distribution of temporal coverage for C1 of full dataset

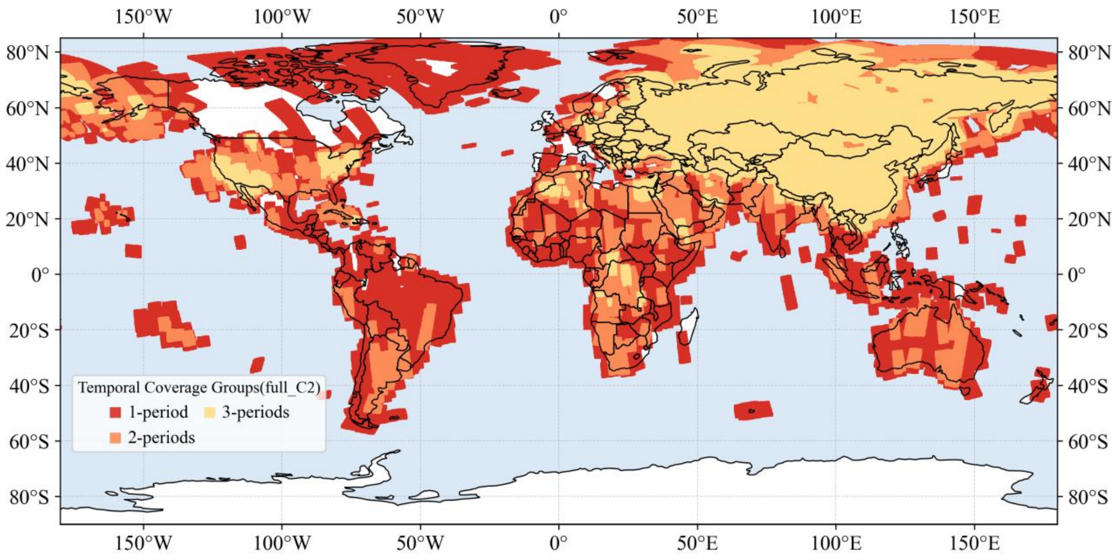

Figure S22. Distribution of temporal coverage for C2 of full dataset

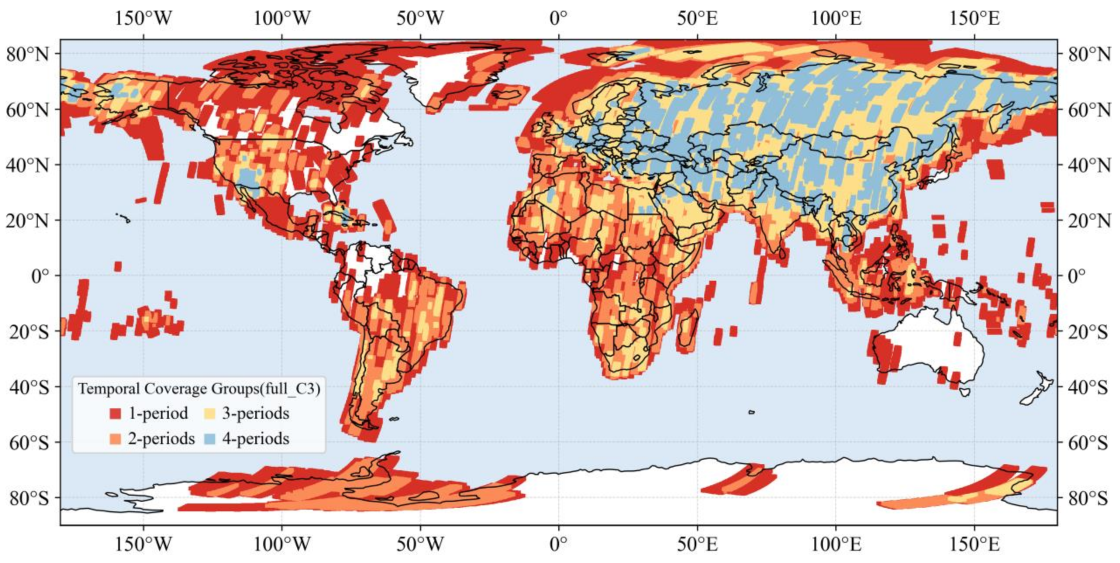

Figure S23. Distribution of temporal coverage for C3 of full dataset
